# Trapped in the Bowel

**DOI:** 10.7759/cureus.19534

**Published:** 2021-11-13

**Authors:** Syed Mustajab Ahmed, Carolina Borz-Baba, Suut Gokturk

**Affiliations:** 1 Internal Medicine, Saint Mary's Hospital, Waterbury, USA

**Keywords:** primary care, right hemicolectomy, intussusception in the elderly, non specific abdominal pain, colonic intussusception

## Abstract

Intussusception in adults is a rare condition and is usually associated with organic disease. It has been implicated for 1% of all bowel obstructions. Clinical presentation can be non-specific and the rarity of the classic triad of abdominal pain, vomiting, and currant jelly stools contributes to late diagnosis and treatment. A 95-year-old lady presented to the emergency department for evaluation of nausea, vomiting, and a two-month history of intermittent diarrhea, which had been worsening for a few days prior to admission. On examination, the abdomen was soft but tender on deep palpation, with audible bowel sounds. No organomegaly or costovertebral angle (CVA) tenderness was appreciated. CT abdomen revealed a long segment of the colon with a loop within loop appearance from the proximal transverse colon to the distal descending colon, consistent with intussusception. The patient was taken to the operating room where local exploration using laparoscopy revealed complete telescoping and intussusception of terminal ileum into the distended ascending and transverse colon and the patient underwent right hemicolectomy. The signs and symptoms of intussusception among the elderly are very non-specific and include nausea, vomiting, change in bowel habits, and gastrointestinal bleeding. Since the classic triad of symptoms (abdominal pain, vomiting, and currant jelly stools) is rarely observed, timely diagnosis and management become a challenge for clinicians. Literature suggests that up to 90% of adults with intussusception present with ongoing abdominal pain. Especially in outpatient settings, patients presenting with intermittent abdominal pain that resolves quickly with simple analgesia should be promptly evaluated. This case illustrates that the rarity of incidence and non-specific clinical presentation are potential barriers towards timely diagnosis and treatment of intussusception among adults, especially the elderly population. Keeping a low threshold for prompt evaluation using appropriate imaging modalities can help overcome this challenge and help reduce the surgical burden.

## Introduction

Intussusception in adults is a rare condition and usually is associated with a structural etiology [1,2]. It has been implicated for 1% of all bowel obstructions [2]. The clinical presentation can be non-specific and the rarity of the classic triad of abdominal pain, vomiting, and bloody stools contributes to late diagnosis and treatment [1], which can potentially lead to life-threatening complications such as strangulation and bowel ischemia. We present a case of a 95-year-old lady presenting with chronic abdominal pain, who was found to have intussusception of a long colonic segment, highlighting the importance of prompt recognition, relevant workup, and treatment options of intussusception.

## Case presentation

A 95-year-old lady with a past medical history of heart failure with reduced ejection fraction (HFrEF) and biventricular implantable cardioverter-defibrillator (ICD), hypertension, and asthma presented to the emergency department for evaluation of nausea, vomiting, and a two-month history of intermittent diarrhea, which had been worsening for a few days prior to admission. Diarrhea was associated with severe, diffuse, waxing, and waning abdominal cramps, which were noted to improve after emesis. No correlation was noted with eating habits and no history of recent antibiotic use was reported. On examination, the abdomen was soft but tender on deep palpation, with audible bowel sounds. No organomegaly or costovertebral angle (CVA) tenderness was appreciated.

**Figure 1:**
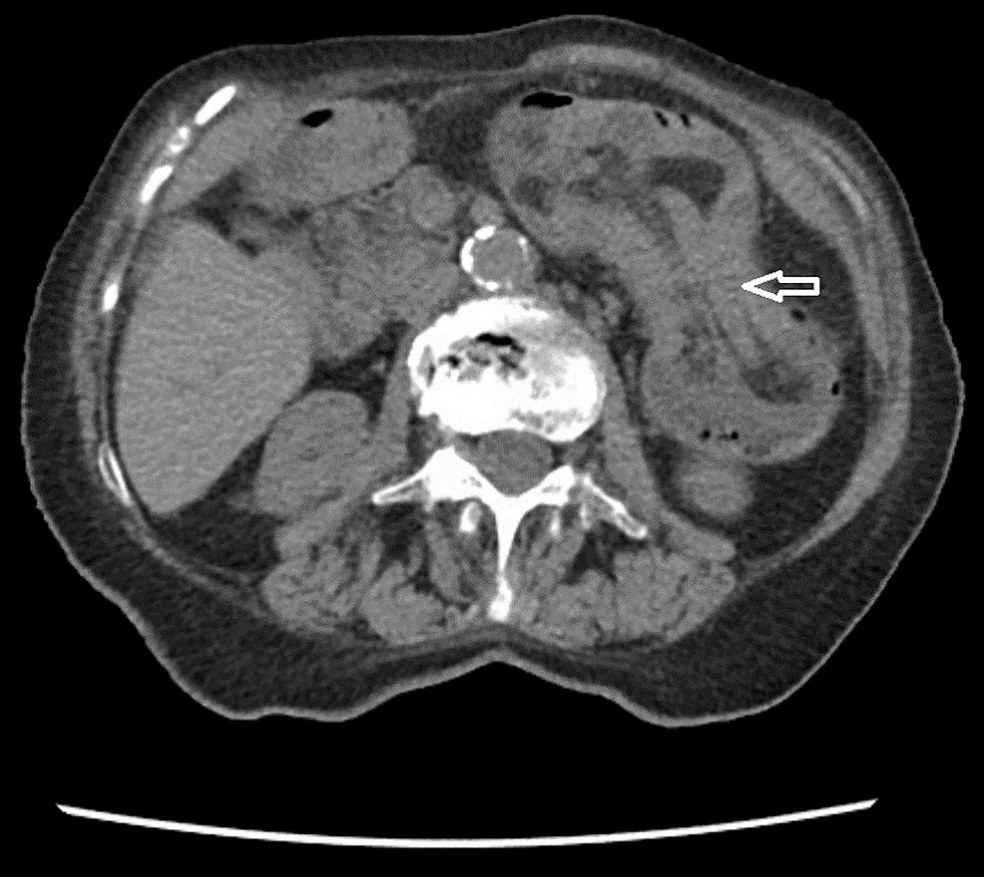
Transverse view of intussusception. The transverse view demonstrates a long segment fat density in the lumen, from the proximal transverse colon to the distal descending colon, consistent with intussusception.

**Figure 2:**
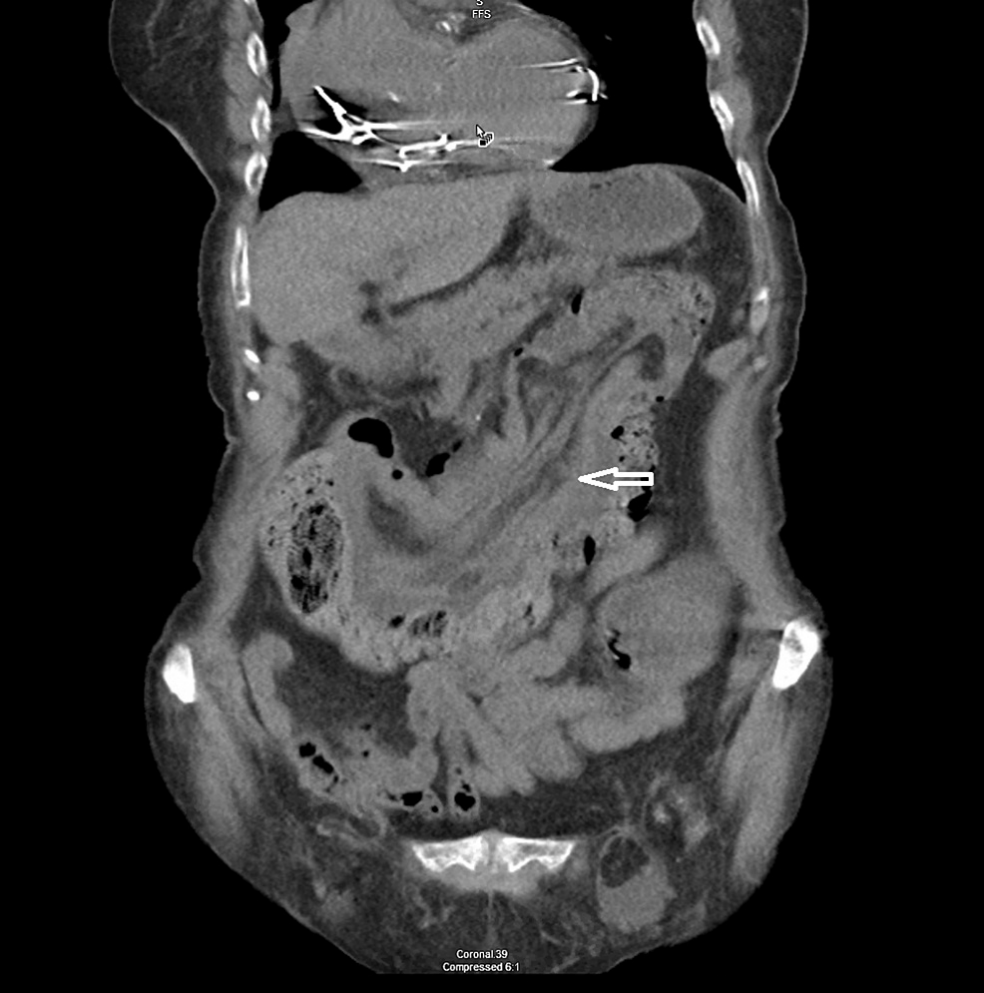
Coronal view of intussusception. The coronal view above demonstrates a long segment fat density in the lumen, from the proximal transverse colon to the distal descending colon, consistent with intussusception.

Initial laboratory results revealed hypokalemia (3.3 mEq/L), lipase within normal limits (32 U/L), and normal transaminases (aspartate aminotransferase [AST]: 19 U/L; alanine aminotransferase [ALT]: 10 U/L) and bilirubin (total bilirubin: 0.7 mg/dl; direct bilirubin: 0.1 mb/dl). Stool studies were unremarkable and coronavirus disease 2019 (COVID-19) reverse transcription-polymerase chain reaction (RT-PCR) was negative. CT abdomen revealed a long segment of the colon with a loop within loop appearance from the proximal transverse colon to the distal descending colon, consistent with intussusception (Figures 1, 2). The underlying lesion for the lead point was not well identified.

The patient was taken to the operating room the next day where local exploration using laparoscopy revealed complete telescoping and intussusception of terminal ileum into the distended ascending and transverse colon. At that time, it was decided to proceed with an open procedure and right hemicolectomy extending from the terminal ileum to about one-third of proximal transverse attachment of the mid transverse colon was performed and side-side anastomosis was created between terminal ileum and transverse colon at the anti-mesenteric border. The patient tolerated the procedure well with no immediate complications. Surgical pathology revealed a tubulovillous adenoma with high-grade dysplasia as the lead point of the intussusceptum. It was negative for in-situ or invasive carcinoma. The postoperative course was significant for acute kidney injury (Cr 1.3 mg/dl with oliguria) on postoperative day two, which resolved after administration of IV fluids. The patient was discharged home with home care services on postoperative day six.

## Discussion

First described by Paul Barbette, intussusception occurs when a part of the gastrointestinal tract invaginates into a neighboring portion of the bowel (intussusceptum), leading to a layer of edematous bowel to form on the outside (intussuscipiens) [1]. It is a rare condition among adults and carries an incidence of 2-3/1,000,000 every year and accounts for 1% of all bowel obstructions.

Most of the cases are associated with an organic etiology, such as malignant tumors in 19-42% and benign tumors in 22-41% of cases [2]. Other etiologies include diverticulosis and postoperative etiologies such as anastomosis and adhesions.

The signs and symptoms are very non-specific and include nausea, vomiting, change in bowel habits, and gastrointestinal bleeding. Since the classic triad of symptoms (abdominal pain, vomiting, and currant jelly stools) is rarely observed in adults, timely diagnosis and management become a challenge for clinicians [1,3,4]. In a case series by Azar and Berger [5], it was reported that 50% of the patients presented to the operating room with a preoperative diagnosis of bowel obstruction, and intussusception was discovered as an intraoperative finding. Literature suggests that up to 90% of adults with intussusception present with ongoing abdominal pain. Especially in outpatient settings, patients presenting with intermittent abdominal pain that resolves quickly with simple analgesia should be promptly evaluated.

Although a number of imaging modalities are available for the evaluation of intussusception, a CT scan has been proven to be the most accurate [6]. Other useful methods include X-ray, ultrasound, barium studies, angiography, and radio-nucleotide studies. The pathognomonic findings observed on CT scan include bowel edema secondary to the telescoping of the proximal intestinal segment into the distal portion, mesentery in the lumen resulting from mesenteric fat transferring along the intussuscipiens, and the classic “sausage sign” and “target sign,” which result secondary to bowel wall thickening and entrapped mesenteric fat [1,4]. In addition, a CT scan can also help localize potential etiology such as mass, polyp, appendix, or diverticulosis.

Colonoscopy can help visualize most of the lead points in ileocolic, colocolic, and sigmoidorectal intussusceptions. This also helps target surgical approach towards specific situations such as appendectomy, polypectomy, or diverticulectomy, leading to uncompromised bowel after the surgery [4].

## Conclusions

In conclusion, the rarity of incidence and non-specific clinical presentation are potential barriers to timely diagnosis and treatment of intussusception among adults, especially the elderly population. Although the incidence is low, intussusception can potentially lead to life-threatening complications. The features of intussusception can be appreciated on different imaging modalities, such as ultrasound and barium enema, but CT scan is most sensitive in diagnosing and planning the treatment strategy. Keeping a low threshold for prompt evaluation using appropriate imaging modalities can help overcome this challenge and help reduce the morbidity and mortality of patients with intussusception.
